# Protocol for a randomized controlled trial of pre-pregnancy lifestyle intervention to reduce recurrence of gestational diabetes: Gestational Diabetes Prevention/Prevención de la Diabetes Gestacional

**DOI:** 10.1186/s13063-021-05204-w

**Published:** 2021-04-07

**Authors:** Suzanne Phelan, Elissa Jelalian, Donald Coustan, Aaron B. Caughey, Kristin Castorino, Todd Hagobian, Karen Muñoz-Christian, Andrew Schaffner, Laurence Shields, Casey Heaney, Angelica McHugh, Rena R. Wing

**Affiliations:** 1 Department of Kinesiology & Public Health, Center for Health Research, California Polytechnic State University, San Luis Obispo, CA USA; 2 Department of Psychiatry and Human Behavior, Alpert Medical School of Brown University, Providence, RI USA; 3 Department of Obstetrics and Gynecology, Alpert Medical School of Brown University, Providence, RI USA; 4 Department of Obstetrics and Gynecology, Oregon Health & Science University, Portland, OR, USA; 5 Sansum Diabetes Research Institute, Santa Barbara, CA USA; 6 Department of World Languages and Cultures, California Polytechnic State University, San Luis Obispo, CA USA; 7 Statistics Department, California Polytechnic State University, San Luis Obispo, CA USA; 8 Dignity Health, Marian Regional Medical Center, Santa Maria, CA USA; 9 Weight Control and Diabetes Research Center, The Miriam Hospital, Providence, USA; 10 Department of Psychiatry and Human Behavior, Alpert Medical School of Brown University, Providence, USA

**Keywords:** Gestational diabetes, Preconception weight loss, Lifestyle intervention, Randomized controlled trial

## Abstract

**Background:**

Gestational diabetes mellitus (GDM) is associated with several maternal complications in pregnancy, including preeclampsia, preterm labor, need for induction of labor, and cesarean delivery as well as increased long-term risks of type 2 diabetes, metabolic syndrome, and cardiovascular disease. Intrauterine exposure to GDM raises the risk for complications in offspring as well, including stillbirth, macrosomia, and birth trauma, and long-term risk of metabolic disease. One of the strongest risk factors for GDM is the occurrence of GDM in a prior pregnancy. Preliminary data from epidemiologic and bariatric surgery studies suggest that reducing body weight before pregnancy can prevent the development of GDM, but no adequately powered trial has tested the effects of a maternal lifestyle intervention before pregnancy to reduce body weight and prevent GDM recurrence.

**Methods:**

The principal aim of the Gestational Diabetes Prevention/Prevención de la Diabetes Gestacional is to determine whether a lifestyle intervention to reduce body weight before pregnancy can reduce GDM recurrence. This two-site trial targets recruitment of 252 women with overweight and obesity who have previous histories of GDM and who plan to have another pregnancy in the next 1–3 years. Women are randomized within site to a comprehensive pre-pregnancy lifestyle intervention to promote weight loss with ongoing treatment until conception or an educational control group. Participants are assessed preconceptionally (at study entry, after 4 months, and at brief quarterly visits until conception), during pregnancy (at 26 weeks’ gestation), and at 6 weeks postpartum. The primary outcome is GDM recurrence, and secondary outcomes include fasting glucose, biomarkers of cardiometabolic disease, prenatal and perinatal complications, and changes over time in weight, diet, physical activity, and psychosocial measures.

**Discussion:**

The Gestational Diabetes Prevention /Prevención de la Diabetes Gestacional is the first randomized controlled trial to evaluate the effects of a lifestyle intervention delivered before pregnancy to prevent GDM recurrence. If found effective, the proposed lifestyle intervention could lay the groundwork for shifting current treatment practices towards the interconception period and provide evidence-based preconception counseling to optimize reproductive outcomes and prevent GDM and associated health risks.

**Trial registration:**

ClinicalTrials.gov NCT02763150. Registered on May 5, 2016

## Administrative information

The order of the items has been modified to group similar items.

**Table:** 

**Title {1}**	Protocol for a randomized controlled trial testing pre-pregnancy lifestyle intervention to reduce recurrence of gestational diabetes: Gestational Diabetes Prevention/Prevención de la Diabetes Gestacional
**Trial registration {2a and 2b}**	ClinicalTrials.gov Identifier: NCT02763150
**Protocol version {3}**	February 1, 2021; version 1
**Funding {4}**	This study is funded by NIH grant R01HD084282
**Author details {5a}**	Suzanne Phelan, PhD. Department of Kinesiology & Public Health, Center for Health Research, California Polytechnic State University, San Luis Obispo, California, USA; sphelan@calpoly.edu Elissa Jelalian, PhD. Department of Psychiatry and Human Behavior, Alpert Medical School of Brown University, Providence, RI, USA; Elissa_Jelalian@brown.eduedu Donald Coustan, M.D. Department of Obstetrics and Gynecology, Alpert Medical School of Brown University, Providence, RI, USA; Donald_Coustan@brown.edu Kristin Castorino, D.O. Sansum Diabetes Research Institute, Santa Barbara, CA, USA kcastorino@sansum.org Aaron B. Caughey, M.D., PhD. Department of Obstetrics and Gynecology, Oregon Health & Science University; Portland, OR, USA; caughey@ohsu.edu Laurence Shields, M.D. Dignity Health, Marian Regional Medical Center, Santa Maria CA;, Laurence.Shields@dignityhealth.org Todd Hagobian, PhD.; Department of Kinesiology & Public Health, Center for Health Research, California Polytechnic State University, San Luis Obispo, California, USA; thagobia@calpoly.edu Karen Muñoz-Christian, PhD; Department of World Languages and Cultures, California Polytechnic State University, San Luis Obispo, California, USA; kschrist@calpoly.edu Andrew Schaffner, PhD; Statistics Department, California Polytechnic State University, San Luis Obispo, California, USA; aschaffn@calpoly.edu Casey Heaney, MS; Department of Kinesiology & Public Health, Center for Health Research, California Polytechnic State University, San Luis Obispo, California, USA; heaney@calpoly.edu Angelica McHugh, M.Ed; Weight Control and Diabetes Research Center, The Miriam Hospital; mchugh1@lifespan.org Rena R Wing, PhD.; Department of Psychiatry and Human Behavior, Alpert Medical School of Brown University; Weight Control and Diabetes Research Center, The Miriam Hospital; rwing@lifespan.org Corresponding Author: Suzanne Phelan, PhD; Department of Kinesiology & Public Health, Center for Health Research, California Polytechnic State University, San Luis Obispo, California, USA; 805 756 2087; sphelan@calpoly.edu.
**Name and contact information for the trial sponsor {5b}**	Eunice Kennedy Shriver National Institute of Child Health and Human Development; NICHDInformationResourceCenter@mail.nih.gov; USA; 1-800-370-2943
**Role of sponsor {5c}**	The NIH was not involved in the study design; collection, management, analysis, and interpretation of data; writing of the report; and the decision to submit the report for publication

## Introduction

### Background and rationale {6a}

Gestational diabetes mellitus (GDM) is a common complication of pregnancy that affects an estimated 7.6% of pregnant persons in the USA [1]. Women with GDM have increased risks for preeclampsia, preterm labor, need for induction of labor, and cesarean delivery as well as increased long-term risks of type 2 diabetes, metabolic syndrome, renal disease, and cardiovascular disease (CVD). An estimated 15–25% of women with prior GDM will develop type 2 diabetes within 1–2 years after pregnancy [2–5], and 35–70% will develop type 2 diabetes 10–15 years after pregnancy [6–9]. Intrauterine exposure to maternal GDM conveys a high risk of several short- and long-term health problems in the offspring and may perpetuate a cycle of obesity [10–12]. Exposure to GDM has been associated with birth trauma, respiratory distress syndrome, neonatal hypoglycemia, and death [13, 14]. GDM increases the risk of excess fetal growth in utero [15], higher infant fat mass [16], neonatal macrosomia, and greater childhood prevalence of obesity (> 90th percentile) through adolescence [17].

One of the strongest risk factors for GDM is the occurrence of GDM in a prior pregnancy. Between 40 and 73% of women with prior GDM will experience GDM recurrence [18–27]. Women with prior GDM have a 3- to 10-fold increased risk of having GDM in a subsequent pregnancy [28, 29]. Women with additional pregnancies complicated by GDM experience threefold increases in the risks of prenatal and perinatal complications and long-term risks of type 2 diabetes, metabolic syndrome, renal disease, and CVD [30–32].

Promising, preliminary research from epidemiologic and retrospective bariatric surgery studies suggests that reductions in body weight before pregnancy may hold the key to the prevention of GDM recurrence [33–39]. Emergent research suggests that it is feasible to recruit women before pregnancy and promote significant weight loss prior to conception [40, 41]. A lifestyle intervention before pregnancy in women with prior GDM may capitalize on a “teachable moment” when women appear more motivated to engage in behavior changes to prevent the recurrence of GDM in a subsequent pregnancy [42–45]. However, an adequately powered randomized clinical trial to test the effects of maternal lifestyle intervention before pregnancy to reduce body weight and prevent GDM recurrence has never been conducted.

### Risk factors for gestational diabetes

While prior GDM is perhaps the strongest risk factor for recurrence of GDM, maternal obesity is also strongly associated with developing GDM during pregnancy [35, 46]. Overweight and obesity affect an estimated 66% of adult women [47], and an estimated 5–12% of women with obesity develop GDM during pregnancy compared with 1–3% of women with normal weight [48]. In epidemiologic studies, the risk of GDM has been four to eight times higher in women with overweight/obesity than with normal weight [48]. A meta-analysis concluded that for every 1 kg increase in pre-pregnancy BMI, the prevalence of GDM was increased by 0.92% [49]. A BMI greater than 35 increases the risk of GDM by about 6-fold [29]. Independent of GDM, maternal obesity is also associated with several other adverse pregnancy outcomes, including preeclampsia, stillbirth, fetal macrosomia, cesarean delivery, and post-surgical wound infection [50, 51]. Obesity is one of the few modifiable risk factors for GDM.

Other risk factors for GDM have been reported in observational [52, 53] and clinical trial [54] studies and include maternal age > 35 [29] (5- to 6-fold increased risk) [54], having a first-degree relative with diabetes (2- to 3-fold increased risk) [28, 29, 54–56], fasting blood glucose of 100–125 mg/dl (7-fold increased risk) [28, 55, 56], HbA1c between 5.8 and 6.4 (5- to 8-fold increased risk) [29, 54], and previous infant with macrosomia (3- to 4-fold increased risk) [53, 54].

Race/ethnicity is another consistent predictor of GDM. People who report Hispanic ethnicity or Native American, Asian, and African-American race have consistently been found to have an increased risk of GDM and recurrent GDM compared with non-Hispanic white women [19, 57–59]. The reasons for the higher prevalence of GDM in non-white women remain unclear. Possible reasons include acculturation among migrant populations and greater exposures to stress, the obesogenic environment, high-energy-dense foods, and obesity [59].

### Prenatal and postpartum interventions to reduce gestational diabetes and related risk factors

Several trials have tested interventions during pregnancy to reduce the incidence of GDM [54, 60–65]. A variety of prenatal interventions have been tried, including approaches that target lifestyle [54, 64], exercise [61, 65], dietary supplementation [60], and/or metformin [66]. Although these trials found positive effects on reducing weight gain during pregnancy, they showed no significant effects on reducing GDM incidence. A network meta-analysis of 23 studies concluded that interventions to prevent the development of GDM were not effective when applied during pregnancy. GDM prevention interventions that begin during pregnancy may be limited by several factors: (1) low intervention intensity during pregnancy out of concerns over effects on the growing fetus, (2) biological changes in pregnancy creating added barriers to adherence (e.g., craving, nausea, edema, weight gain), and (3) a very short intervention window (≤ 2 months) prior to GDM diagnosis.

Postpartum diet and exercise interventions in women with prior GDM have also been tested and shown more promise in reducing risk factors for subsequent diabetes and CVD [67–78], although low engagement [79], modest effects on weight [80], and poor adherence remain problematic [81]. The Diabetes Prevention Program included women with previous GDM [74], and consistent with the full sample results, lifestyle intervention for weight loss or metformin significantly reduced the incidence of diabetes by 50% compared with the placebo group [74, 82]. While postpartum intervention in women with prior GDM reduces the risk of diabetes, the effects of postpartum intervention on recurrence of GDM in a subsequent pregnancy and effects on future maternal and child health outcomes have not been investigated. Intervening in the immediate postpartum period to help women lose weight may be too distal from subsequent pregnancy to exert a protective effect on GDM recurrence, but clinical trial data are lacking.

### Preconception weight loss to reduce gestational diabetes and associated health risks

Observational research suggests that women who experience even modest weight losses (> 10 pounds) [33, 34, 83] or less weight gain [84–86] prior to pregnancy significantly reduce their risk of GDM development compared to women who maintain weight or gain > 10 pounds. Kim et al. [48] estimated that up to half of GDM cases could be prevented by reducing pre-pregnancy obesity. Retrospective data from bariatric surgery populations also suggest that weight loss in women with obesity prior to pregnancy may reduce the risk of GDM and its recurrence [37, 87, 88] and prevent transmission of obesity to children [36]. Other observational research has shown that maternal consumption of healthy food and avoidance of unhealthy foods [89] and engagement in regular physical activity before pregnancy were independently associated with reduced risk of subsequent GDM [89]. Honein et al. [90] estimated that if 10% of women with pre-pregnancy obesity achieved a healthy weight (BMI < 25) before pregnancy, nearly 300 congenial heart defects and 700 fetal deaths per year could be prevented each year.

However, few clinical trials have examined the effects of weight loss during the preconception period on subsequent outcomes [41, 91]. The PREPARE randomized trial [41] tested a phone-based weight loss intervention in 326, non-Hispanic (94%) women with a BMI ≥ 27 and found that, relative to the control group, women in the lifestyle intervention lost more weight prior to conception (3.7 vs. 0.6 kg, respectively). After 24 months, 169 (52%) became pregnant and were included in the analysis. The results indicated that those in the preconception intervention group surprisingly gained more weight during their subsequent pregnancy than those in the control group (13.2 vs. 10.3 kg gain, respectively; *p =* 0.03); there were no significant differences in GDM or other pregnancy outcomes, with the exception of spontaneous pregnancy losses, which were less common in the intervention arm. Participants in the intervention arm had a 10% lower absolute rate of GDM than in the control arm (25% vs. 35%, respectively), but the study had insufficient power to evaluate whether or not a difference of this size was due to chance.

A trial in Finland [91] randomized women with obesity and/or prior GDM to a nurse-led preconception lifestyle intervention or a control group. Among the 65% who became pregnant during the trial, there were no significant differences by randomized group in the cumulative incidence of GDM, which was 60% (*n* = 39/65) in the intervention group and 54% (*n* = 34/63) in the control group (*p* = 0.49). However, 45% of the participants included in the final analyses received only 1 preconception visit or no intervention at all, and thus, preconception weight change was not analyzed [91]. Other studies have demonstrated feasibility [40] and examined the effects in women with fertility issues [92], and other trials are in progress [93]. Clinical trial data are needed to test whether preconception weight loss can prevent GDM recurrence [35, 46].

### Lifestyle interventions based on social-cognitive-theory

Lifestyle interventions based on social cognitive theory (SCT) and “teachable moment” models provide a rich foundation for effective intervention to prevent GDM recurrence. SCT-based interventions have been effective in promoting weight control in a variety of patient populations and treatment modalities, including the DPP program [94, 95]. SCT emphasizes the dynamic interplay of the individual and the environment in adopting behavior changes and posits that a sense of self-efficacy must be developed through the use of self-regulation skills (i.e., goal-setting, self-monitoring, problem-solving, incentives) that foster weight control and healthy eating and physical activity behaviors. SCT-based interventions delivered prior to pregnancy may capitalize on a “teachable moment” for promoting long-term behavior change. “Teachable moments” are naturally occurring life transitions or health events thought to augment motivation for adopting risk-reducing health behaviors [96, 97]. Women who are planning a pregnancy may be more motivated to change their eating and exercise behaviors and lose weight for the health of their pregnancy.

Moreover, focusing on women with a history of GDM may maximize motivation. To date, the trials testing weight loss interventions before pregnancy have focused on a general population of women with obesity. An alternative approach is to focus on women who are at particularly high risk of GDM because they had GDM in a prior pregnancy. Women with prior GDM report high motivation to change behaviors to prevent GDM recurrence and protect the health of their future child [42–45]. A SCT-based lifestyle intervention may capitalize on this motivation and promote significant pre-pregnancy weight loss and maintenance. Optimizing maternal weight and the intrauterine environment before pregnancy holds promise for preventing GDM recurrence and improving short- and long-term maternal/child health. However, no study to date has been designed to test the efficacy of a comprehensive pre-pregnancy lifestyle weight loss intervention to prevent GDM recurrence in a racially/ethnically diverse group of women.

### Potential mechanisms

Lifestyle treatment targeting weight, physical activity, and dietary intake before pregnancy may reduce GDM through effects on insulin and inflammatory factors. While pathogenesis linking pre-pregnancy obesity and GDM remains under investigation, obesity during pregnancy appears to augment a systemic inflammatory response that leads to greater insulin resistance and glucose dysregulation [98]. Both obesity and GDM are associated with increased circulating levels of leptin [99] and the inflammatory markers TNF-alpha [100] and C-reactive protein [98] and decreased levels of adiponectin [101]. Over time, the chronic and acute insulin resistance and inflammation independently associated with obesity and pregnancy, respectively, may lead to a progressive loss of insulin secretion that increases the risk of developing diabetes and other diseases later in life [102]. Clinical trial data are needed to identify the mechanisms most impacted by pre-pregnancy weight loss and linked with prevention of GDM and improved insulin resistance.

### Objectives {7}

The Gestational Diabetes Prevention/Prevención de la Diabetes Gestacional is a two-site randomized clinical trial testing the efficacy of a pre-pregnancy lifestyle intervention to reduce GDM recurrence in women with overweight and obesity. The trial is following the CONSORT guidelines [103]. The primary hypothesis is that the recurrence of GDM will be reduced among participants assigned to pre-pregnancy lifestyle intervention vs. educational control group. Test for GDM will be conducted at 24 to 28 weeks’ gestation. Secondary hypotheses are that the pre-pregnancy lifestyle intervention (vs. educational control group) will result in improved maternal fasting glucose and biomarkers of insulin resistance (insulin, leptin, TNF-alpha, C-reactive protein, and adiponectin) and CVD risk (lipids and blood pressure) assessed before pregnancy, after 16 weeks of intervention, and at 26 weeks’ gestation. The pre-pregnancy lifestyle intervention (vs. educational control group) is expected to reduce adverse perinatal health outcomes for mothers (gestational hypertension, preeclampsia, preterm delivery, excessive gestational weight gain, induction of labor, cesarean delivery) and neonates (admission to the neonatal nursery, hyperbilirubinemia, birth trauma, weight for length *z*-scores ≥ 95% at birth and 6 weeks). Also, the pre-pregnancy lifestyle intervention (vs. educational control group) is hypothesized to result in greater pre-pregnancy weight loss and improvements in diet (calories, % fat, fast food) and physical activity (minutes of moderate activity). In exploratory mediator analyses, treatment-related changes in pre-pregnancy weight, eating, and activity are expected to be related to improvements in maternal physiology and reduced odds of GDM recurrence.

### Trial design {8}

This study is a two-site, parallel-group, randomized clinical trial comparing a pre-pregnancy lifestyle modification intervention vs. educational control. A total of 252 women with overweight or obesity and a history of GDM will be randomized using an allocation ratio of 1:1 to either a pre-pregnancy lifestyle weight loss intervention vs. control condition. This is a superiority trial. Assessments occur before pregnancy (at study entry, after 16 weeks, and brief visits every 16 weeks until conception), during pregnancy (at 26 weeks’ gestation), and at delivery and 6 weeks postpartum.

## Methods: Participants, interventions, and outcomes

### Study setting {9}

The study includes two clinical sites. One site is at California Polytechnic State University, San Luis Obispo, CA (S. Phelan, PI), and the other site is at Brown University and the Miriam Hospital in Providence, RI (R. Wing, PI).

### Source population

GDM recurrence rates are higher in Hispanic and African-American populations [57]; thus, our targeted recruitment plan includes 35% Hispanic, 9% African American, 6% Asian, and 50% non-Hispanic white at each site. The geographical regions and recruitment clinic populations selected for this study have a high prevalence of obesity (≥ 35%) and client diversity (35–45% Hispanic; 9% African American).

### Eligibility criteria {10}

Table 1 describes the eligibility and exclusion criteria for this trial. Participants must have physician documentation of GDM during any prior pregnancy. Given the diversity of clinically acceptable methods used to diagnose GDM [104–107], several diagnostic methods for prior GDM are eligible. Acceptable documentation to confirm prior GDM are as follows: (1) a 3-h 100 g oral glucose tolerance test (OGTT) performed at ≥ 20 weeks’ gestation in which 1 or more values exceeded the Carpenter and Coustan criteria [108] (i.e., fasting ≥ 95 mg/dL; 1 h ≥ 180 mg/dL; 2 h ≥ 155 mg/dL or 3 h ≥ 140 mg/dL), (2) a 75-g OGTT performed at ≥ 20 weeks’ gestation and 1 or more values exceeded the International Association of Diabetes and Pregnancy Study Groups (IADPSG) criteria [109] (i.e., fasting ≥ 92, 1 h ≥ 180; 2 h ≥ 153), (3) a 1-h 50 g test performed at any time during pregnancy with a value of ≥185 mg/dL (if ≥130 mg/dL but < 185 mg/dL and the clinic did not do a follow-up 100-g OGTT, the participant would be ineligible), (4) a fasting glucose value prior to 20 weeks’ gestation was ≥ 92 mg/dL and < 125 mg/dL and treatment with medication or insulin, or (5) an HbA1c conducted anytime during pregnancy with a value of > 5.6% and the treatment with medication or insulin.

**Table 1:** Inclusion and exclusion criteria for Gestational Diabetes Prevention/Prevención de la Diabetes Gestacional

Inclusion criteria	Prior diagnosis of GDM
	Planning to have a baby in the next 1–3 years
	BMI > 25 kg/m^2^
	English- or Spanish-speaking
	Breastfeeding or non-breastfeeding
	Literacy ≥5th grade level
	Access to a cell phone
Exclusion criteria	Age < 18 years
	HbA1c test (> 6.5%)
	≥ 3 months postpartum
	Current pregnancy
	Tubal ligation Semi-permanent form of birth control with no plans for removal (e.g., hormonal progesterone intrauterine device or hormonal contraceptive implant)
	Relocating in the next 2 years
	Medications that affect weight or diabetes (e.g., oral corticosteroid and metformin)
	Use of weight loss medications
	Serious current physical disease (e.g., heart disease, cancer, renal disease, and diabetes) for which physician supervision of diet and exercise prescription is needed
	Orthopedic limitations to aerobic exercise
	History or plans of bariatric surgery
	Current problems with drug abuse and/or symptoms of an eating disorder, which occurred less than 3 years year ago
	Hospitalization for depression or psychological problems in the last year
	No show to a scheduled orientation and fail to reschedule

Women must also report chances of having a baby in the next 1–3 years; this is determined based on a response to the question, “On a scale of 0–10, what are the chances you see yourself ever having any more children?” Participants reporting ≥ 1 on this scale and who report plans for pregnancy within the study’s time frame (1–3 years depending on study enrollment year) are considered eligible. Other eligibility criteria include BMI ≥ 25 kg/m^2^, age ≥ 18 years, and English- or Spanish-speaking. Breastfeeding women are eligible to enroll, as moderate weight loss does not appear to adversely affect lactation [110–114]. At study enrollment, participants are encouraged to use medically proven forms of contraception until completion of the initial 16 weeks of intervention, but this is not an eligibility requirement.

Women with type 2 or type 1 diabetes are excluded; the lack of diabetes is confirmed prior to randomization with an HbA1c test (> 6.5%). Women with a family history of diabetes or with impaired glucose tolerance (“prediabetes”; HbA1c of 5.7–6.4%) may be at increased risk of GDM [115] but are included because weight loss could potentially still modify the risk of GDM. Other exclusions include age < 18 years, current pregnancy, tubal ligation, semi-permanent form of birth control with no plans for removal (e.g., hormonal progesterone intrauterine device or hormonal contraceptive implant), relocating in the next 2 years, medications that affect weight/diabetes (e.g., oral corticosteroid and metformin), serious current physical disease (e.g., heart disease, cancer, renal disease) for which physician supervision of diet and exercise prescription is needed, orthopedic problems that limit the ability to exercise [116], problems with drug abuse and/or symptoms of eating disorders [117], history or plans of bariatric surgery, and hospitalization for depression or psychological problems in the past year. Also, women who do not show to orientation visits and fail to reschedule are excluded.

### Enrollment process and consenting

**Fig. 1:**
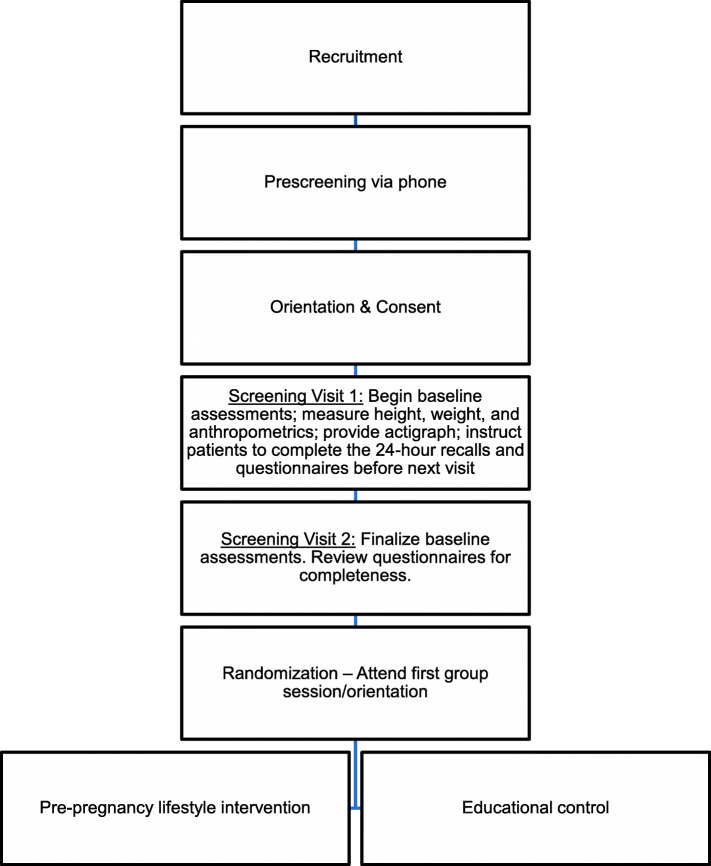
Overview of entry into Gestational Diabetes Prevention/Prevención de la Diabetes Gestacional

Figure 1 shows the process of study enrollment—from recruitment to randomization. Women who appear interested in the program are screened by phone. If still eligible after phone screening, patients are asked to attend an orientation and consenting visit followed by their baseline assessment visit.

### Who will take informed consent? {26a}

Trained study staff collect the informed consent.

### Additional consent provisions for collection and use of participant data and biological specimens {26b}

The process of informed consent includes a discussion of an option to allow the collection and storing of additional biospecimens for future research that may include analyses on genetic material.

## Interventions

### Explanation for the choice of comparators {6b}

Two groups are compared in this study. Group 1 is a standard care plus education control group and was selected because the intervention provides a level of care that is consistent with the typically minimal amount of lifestyle counseling received by women before pregnancy and also is not expected to promote clinically significant weight loss. Group 2 is standard care plus education plus weight loss intervention and was selected in order to isolate the effects of pre-conception weight loss on GDM recurrence and other health outcomes.

### Intervention descriptions {11a}

#### Group 1: Standard care + education

Women in this condition receive usual medical care before and during pregnancy and throughout the trial. Also, these women meet with a study interventionist for 20-min individual sessions at study entry and again after 16 weeks. The first meeting at study entry encourages women to spend the next 16 weeks improving overall health before pregnancy and reviews nutrition (e.g., consuming multivitamins, folic acid) and physical activity recommendations. The second face-to-face meeting occurs after 16 weeks and focuses on managing stress and also includes a *Pregnancy Primer*. Since all women in the study have expressed a desire to have another pregnancy within 1–3 years, this module provides participants with standard information on methods to track ovulation. Participants also receive information on the recommended amount of weight gain [118] during pregnancy. Throughout the study, women receive quarterly study newsletters with study updates and general information about preconception health and wellness.

#### Group 2: Standard care + education + weight loss intervention

##### Overview

This group receives all aspects of group 1 plus a standard lifestyle modification program implemented to induce ≥ 10% weight loss over 16 weeks and promote weight loss maintenance over subsequent months (12–36 months depending on enrollment year) until conception. This comprehensive, individually focused, SCT-based weight control program includes education, behavioral self-regulatory strategies, ongoing contact, feedback, and social support. The intervention is based on the DPP and Look Ahead lifestyle interventions [101, 119, 120], which have been proven effective in promoting significant weight loss and maintenance in multiethnic, English- and Spanish-speaking individuals across the country [119, 120]. The intervention provides guidance and resources for English- and Spanish-speaking individuals from a variety of different cultures and backgrounds.

##### Format and contact

The intervention focuses on ongoing, individual contact with a study interventionist to promote weight loss prior to conception. Visits may be conducted in person, on the phone, or through video conferencing. For the first 16 weeks, participants meet weekly for ~ 30 min. Thereafter, participants meet bi-weekly (or more frequently in the context of weight regain) to maintain weight loss until conception. After conception, intervention contacts are discontinued.

##### Weight loss goals

Participants are given a scale and told to aim for a weight loss of 1–2 lb per week for the first 16 weeks. Patients desiring to lose more weight during the program are encouraged to do so, provided they maintain reasonable eating and activity patterns and do not reduce below normal weight. After 16 weeks, participants may work on weight loss maintenance or continue to lose weight at a moderate rate (1–2 lb/week) until confirmed conception.

##### Dietary goals

Participants are instructed to follow a standard calorie restriction diet used in lifestyle modification programs. Calorie goals are based on study entry weight with 300 cal/day adjustments for breastfeeding status, if applicable. Participants with an entry weight of < 91 kg are prescribed a 1200-kcal/day self-selected diet, and those with an entry weight of > 91 kg are prescribed a 1500-kcal/day. A standard, low-fat diet is prescribed (35% fat, 20% protein, 45% CHO), since prior research has suggested that recurrence of GDM was greater in women who consumed more fat between pregnancies [121]. After the 16-week program, dietary goals may be adjusted to help women maintain their weight loss or weight maintenance goal until conception.

##### Exercise goals

Participants are instructed to increase their physical activity to at least 150 min per week during the initial 16-week program (e.g., 30 min per day, 5 days per week). Thereafter, they are advised of higher goals (60 min/day) to promote long-term weight loss maintenance. Brisk walking, “child-friendly,” and inexpensive activities are suggested, taking into consideration potentially unsafe neighborhoods. Participants are provided with a pedometer and encouraged to gradually increase the number of steps they walk per day (with an increase of ~ 250 steps/day each week) until reaching an ultimate goal of about 10,000 steps per day [122].

##### Behavioral goals

The major features of behavior modification include self-monitoring, behavior chains, stimulus control, goal-setting, self-reinforcement, problem-solving, social assertion, and cognitive strategies [123]. Participants complete weekly behavioral assignments, which are reviewed by the interventionist. Participants are given self-monitoring records or encouraged to use apps, if preferred, to facilitate self-monitoring.

### Criteria for discontinuing or modifying allocated interventions before conception {11b}

Site physicians who are trained in obstetrics/gynecology guide decisions as to whether or not to continue intervention or assessments in women with medical difficulties. If needed, the study physicians contact a participant’s provider to discuss treatment/assessment continuation for participants. The study physicians do not provide medical care during the course of the study but refer and help participants obtain appropriate medical or psychiatric care, if needed. The rate of weight loss and caloric restriction are monitored, and if any extreme and overly rapid weight losses occur, healthier practices are encouraged and adherence to these recommendations is monitored. A subset of women may be breastfeeding upon enrollment. Moderate weight loss does not appear to adversely impact breastfeeding [113, 124], but study staff are monitoring such occurrences and provide referrals, as needed. To reduce the risk of muscle soreness, muscle strain, or joint sprain; loss of balance; or trauma by falling, the physical activity (walking) is moderate, progressive, and volitional, and goals may be modified, as needed for an individual participant.

### Strategies to improve adherence to interventions {11c}

The behavioral weight loss program includes a variety of strategies to improve adherence. These include strategies related to appropriate goal setting, cognitive restructuring, relapse prevention, and problem-solving. In addition, the study interventionist provides support for all positive behavioral changes. Weekly supervision meetings with the intervention team are designed to promote treatment fidelity. Participant cases are reviewed and strategies discussed with the intervention team to promote participant adherence to attending treatment sessions, completing food and exercise records, engaging in daily self-weighing, and following calorie and activity goals. Intervention fidelity measures (coded audiotaped sessions) are further reviewed and discussed to guide and promote adherence.

### Relevant concomitant care permitted or prohibited during the trial {11d}

During the trial, participants are not restricted from receiving concomitant care and/or interventions.

### Provisions for post-trial care {30}

There is no provision for post-trial care. There is no anticipated harm and no compensation for harm due to trial participation.

### Outcomes {12}

#### Primary outcome

The study’s primary outcome is GDM diagnosis in the next pregnancy. The 2-step approach of diagnosing GDM is done at 24–28 weeks’ gestation and involves participants receiving as part of standard care a 50-g oral glucose solution followed by a 1-h venous glucose determination. Participants meeting or exceeding the 1-h screening criteria (a cutoff for an abnormal 1-h screen of ≥ 130 mg/dL) are then referred to the study for completion of the 100-g, 3-h diagnostic oral glucose tolerance test (OGTT). Positive diagnosis of GDM is based on the Carpenter and Coustan criteria [108] based on 2 abnormal values on the 3-h OGTT that includes a fasting value of > 95 mg/dL, 1 h > 180 mg/dL, 2 h > 155 mg/dL, 3 h > 140 mg/dL. The results of the study’s 100-g OGTT are immediately shared with the participants’ medical providers who interpret and inform patients of GDM screening results. If the study measured 100-g, 3-h diagnostic OGTT is not obtained, provider assessments done in standard care that are based on acceptable diagnostic methods (Table 2) are used to diagnose GDM. Final determination of GDM diagnosis is done by an independent evaluation of records from the study’s Ob/Gyn physician-researchers who are masked to the randomized group.

**Table 2:** Diagnostic criteria for GDM in Gestational Diabetes Prevention/Prevención de la Diabetes Gestacional

1.	3-h 100 g OGTT performed at ≥ 20 weeks’ gestation in which 2 or more values exceed the criteria as follows: fasting ≥ 95 mg/dL; 1 h ≥ 180 mg/dL; 2 h ≥ 155 mg/dL or 3 h ≥ 140 mg/dL [108]
2.	75 g OGTT performed at ≥ 20 weeks’ gestation and 1 or more values exceed (fasting ≥ 92, 1 h ≥ 180; 2 h ≥ 153) [109]
3.	1-h 50 g test performed at any time during pregnancy with a value ≥ of 200 mg/dL
4.	Fasting glucose value prior to 20 weeks’ gestation ≥ 92 mg/dL and < 125 mg/dL and clinic treats the patient for GDM with medication or insulin
5.	HbA1c conducted anytime during pregnancy with a value ≥ of 5.7% and the clinic treats the patient for GDM with medication or insulin

#### HbA1c tests

Diabetes is an exclusion criterion (based on HbA1c at screening > 6.5%). However, after screening, annual HbA1c tests are performed until conception and test results shared with the participants’ providers. This is expected to minimize early clinical screenings for pre-existing DM; however, any participant receiving an early (< 24 weeks’ gestation) clinical diagnosis of GDM is included in the analysis.

#### Maternal insulin resistance/physiologic parameters

Maternal fasting glucose, insulin, homeostatic model assessment of insulin resistance (HOMA-IR), leptin, TNF-alpha, C-reactive protein, adiponectin, and lipids are measured by trained staff at each research site, following the established protocols. Blood draws are scheduled 12–24 h after the most recent bout of exercise and after an overnight fast. HOMA-IR is used to estimate insulin resistance. Systolic and diastolic blood pressure are measured using a standard mercury manometer and appropriate size cuffs with participants in the sitting position with both feet on the ground. After resting for 5 min, the average of two measurements is recorded, with a 1–2 min interval between measures.

#### Maternal anthropometrics

Weight is measured to the nearest 0.1 kg using calibrated standard digital scales. Two measures are completed with participants measured in light clothing (without shoes). Scale calibration is checked weekly with known weights. Standing height is measured twice in patients without shoes in millimeters with wall-mounted Harpenden stadiometers. Given the emerging evidence of a relationship between abdominal fat and GDM [125], waist circumference is measured over bare skin or underwear using a tape measure and following standardized protocols.

*Lifestyle behaviors* are measured to examine the treatment effects and relationships with maternal physiology and GDM recurrence. Physical activity is measured for 7 days using the actigraph accelerometer (MTI, Inc.) which provides minutes and time spent in light, moderate, and vigorous activity over a period of days or weeks [126–128]. TV and sedentary behavior are assessed by pre-established questionnaires [129, 130]. Dietary intake is measured using 24-h recalls on 2 random days over a week [131–135] and completed in an interview format using the NCI Automated Self-Administered 24 h recall (ASA24 http://riskfactor.cancer.gov/tools/instruments/asa24.html). The primary variables of interest are calories, protein, carbohydrates, and fat; consumption of sugar-sweetened beverages; and fast food. Fast food consumption is also assessed based on validated self-report questions [136]. Weight control practices are assessed using the validated Weight Control Strategies Scale [137]. A supplemental brief assessment [138] is administered to assess the frequency of self-weighing, self-monitoring, and meal patterns. Given the association between perceptions of risk and adoption of lifestyle changes, the perceived risk of GDM recurrence is measured at baseline and after 4 months using the Risk Perception Survey modified for GDM [139]. The Center for Epidemiologic Studies Depression (CES-D) screener [140] is used to examine the levels of depressive symptoms; the 14-item Perceived Stress Scale is used to measure the levels of stress, which is a predictor of GDM [141]; and the Eating Inventory [142] assesses the three dimensions of dietary restraint, including cognitive restraint, disinhibition, and hunger. The General Sleep Disturbance Scale (GSDS) is used for a subjective measure of sleep disturbance which has been related to the risk of GDM [143].

#### Maternal/infant consequences of GDM

Chart abstractions are conducted by trained research staff to determine whether the intervention results in fewer adverse maternal and neonatal health outcomes. Consistent with prior research, rates of inadequate and excessive GWG are computed based on the National Academy of Medicine guidelines [118], using measured pre-pregnancy and last clinic visit weights. Other maternal adverse outcomes clinically defined based on chart abstractions include gestational hypertension, preeclampsia, cesarean delivery, labor induction, and delivery < 37.0 weeks’ gestation. Adverse outcomes among infants include birth weight greater than 4000 g, large size for gestational age (defined as birth weight above the 90th percentile), and small size for gestational age (birth weight below the 10th percentile) [144]. A composite measure of serious perinatal complications is defined as one or more of the following: death (stillbirth or neonatal death), hypoglycemia, hyperbilirubinemia, neonatal hyperinsulinemia, shoulder dystocia/birth trauma (brachial plexus palsy or clavicular, humeral, or skull fracture), admission to the neonatal intensive care unit (NICU), and respiratory distress syndrome [13, 145]. An additional composite morbidity outcome is computed based on prior research in women with obesity that includes at least one of the following: cesarean delivery, hypertensive disorders of pregnancy (HDP), birth weight ≥ 4000 g, birth weight < 2500 g, or NICU admission [146]. In women diagnosed with GDM, prescribed treatments are examined to explore whether the intervention impacted the intensity of treatment/severity of GDM.

#### Infant measures

At 6 weeks, infant length, weight, and skinfold thickness measurements are performed by trained staff using standardized procedures. BMI *z*-scores are calculated using the WHO Child Growth Standards for age and sex [147]. A *z*-score of > 1 will be used to define at risk for obesity.

#### Demographics and medical/reproductive history

At baseline, participants complete a demographic questionnaire assessing age, race, ethnicity, and weight history (e.g., inter-pregnancy weight changes). Given prior relationships with GDM recurrence [148], extensive pregnancy history information (maternal and neonatal) are collected [149, 150]. At follow-ups, changes in smoking, prescription medications, unsafe dieting practices [117], job status, and participation in other weight loss programs, and changes in medical history are assessed. Pregnancy urine tests are used to assess pregnancy status at quarterly visits until conception. Pregnancy confirmation is documented through clinical ultrasound results.

#### Process measures

Recruitment, eligibility, refusal rates/reasons, and retention rates/reasons are tracked, as well as the number of women who conceive during the trial and the average duration until conception. Intervention acceptability is measured based on participants’ ratings of various aspects of the program, the interventionist, content, and overall impression. To measure intervention fidelity, all intervention sessions are audiotaped, and a randomly selected subset (10%) is coded for content by a trained study staff (not involved in any assessment data collection). Adherence to the intervention is measured via attendance at treatment sessions, number of self-monitoring records returned, and the activity, eating, and behavioral measures. To assess the safety of the intervention, levels of hunger, depressive symptoms, injuries due to physical activity, changes in medical status, and unintended reduction in milk supply (in breastfeeding women) are assessed.

### Participant timeline {13}

Table 3 shows when participants complete the measures in Gestational Diabetes Prevention/Prevención de la Diabetes Gestacional. Bilingual (English/Spanish-speaking) assessors are masked to randomization and conduct all major assessments occurring at baseline, after 16 weeks, at 26 weeks’ gestation, and at 6 weeks postpartum. These assessments occur at the study’s research centers or affiliate locations most proximal to participants. If necessary, assessments are conducted at participants’ homes. After 16 weeks, quarterly brief assessments occur until conception. The brief assessments may be conducted in person or over the phone/video conferencing.

**Table 3:** Measures in Gestational Diabetes Prevention/Prevención de la Diabetes Gestacional

	Assessment time point				
	Pre-pregnancy			Pregnancy	Postpartum
	Baseline	4 months	Every 4 months until pregnancy	26 weeks’ gestation	6 weeks
Demographics and medical history	X	X	–	X	X
Weight, height, waist circumference	X	X	X	X	X
Pregnancy test	X	X	X	–	–
**Maternal physiology**					
HbA1c (screening then annually until conception)	X	–	–	–	–
GDM assessment	–	–	–	X	–
Glucose, insulin	X	X	–	X	–
Inflammatory factors	X	X	–	X	–
Lipids	X	X	–	X	–
Blood pressure	X	X	–	X	–
**Lifestyle behaviors**					
Physical activity	X	X	–	X	–
Dietary intake	X	X	–	X	–
Behavioral and psychosocial	X	X	brief	X	–
**Infant weight, length**	–	–	–	–	X
**Maternal/infant complications**	–	–	–	–	X
**Process measures**	–	X	X	–	–

### Sample size {14}

Power calculations assume a 60% GDM recurrence rate in women with overweight or obesity [19, 57, 58]. With a target sample size of 252 participants and assumed ≥ 70% pregnancy rate [151, 152] and 30% lost to follow-up or not pregnant before conception, ≥ 176 pregnant participants (≥ 88 in each group) would provide adequate statistical power (≥ 81.98%) to detect intervention effects on the proportions developing recurrent GDM [83, 153], taking into account estimations of site-specific clustering effects and effect modifiers (i.e., weight status, ethnicity, parity) of GDM recurrence [24, 57]. Under this scenario, the minimum detectable effect size (odds ratio) would be 0.43, and proportions with GDM in educational control and intervention groups respectively would be 60% (*n* = 53/88) and 38% (*n* = 33/88). For secondary aims, 88 pregnancies in each group would yield > 80% power to detect effects equal to or smaller than those reported in prior work testing effects of lifestyle interventions on reductions in glucose, triglycerides, CRP [154], insulin, leptin [155], adiponectin [156], and blood pressure [157], taking into account estimations of site-specific clustering effects and effect modifiers (i.e., weight status, ethnicity, parity) of insulin resistance [158], and CVD risk factors [159, 160]. For secondary aims examining effects of the intervention on prenatal and perinatal complications, the study would have > 80% power to detect moderate effect sizes [161]. For the fourth aim examining the effects of the intervention on pre-pregnancy weight losses and improvements in eating (calories, macronutrient balance, fast food) and activity, 88 participants in each group would yield > 90% power to detect effects reported in prior work testing effects of lifestyle interventions on reductions in weight [157] and behavioral variables [162, 163]. For exploratory mediator analyses, > 80% power is achieved to detect > 21% increase in the mediated odds of GDM per kilogram difference in pre-pregnancy weight loss (equivalent to a mediated slope in a logistic model of > 0.189).

### Recruitment {15}

To reach the target sample size of 252, a 3-year recruitment time frame is proposed. Both sites recruit participants through direct and indirect methods and via administrative databases. As described in Table 4 direct recruitment methods include clinic staff and research assistants at the recruitment clinics providing information about the study at the time of prenatal or postnatal visits for patients with GDM or prior GDM. Indirect methods include presentations in healthcare settings that interact with mothers (e.g., Ob/Gyn, pediatrician, WIC offices). Administrative databases are also used to identify and offer the program to women with a history of GDM who might not regularly engage with the targeted healthcare settings after having a baby.

**Table 4:** Recruitment methods for reaching preconception women with prior gestational diabetes mellitus

**Direct to patient**	
In-person recruitment by clinic or study staff during pregnancy or 6 weeks postpartum visits	
Brochures and posters in clinics and waiting rooms	
Social media (Facebook, Instagram) posts, videos, and advertisements	
Traditional media (television, radio, flyers)	
Online forums posts	
**Indirect through women’s health providers (e.g., Ob/Gyn, WIC)**	
Presentation and clinic meetings	
1-page fact sheets	
Study write-up for newsletters directed at providers	
**Administrative databases**	
Hospital patient databases	
Community health center patient database	
Review of research center database	
Research match	

## Assignment of interventions: allocation

### Sequence generation {16a}

Eligible participants are randomized in a 1:1 ratio into the intervention or control group based on a computer-generated (R 4.0.4 for Windows) random sequence. Randomization is stratified by site, pre-diabetes status (HbA1c < 5.7 vs. ≥ 5.7), and prior method of GDM diagnosis (one-step, 2 h test vs. other methods) to ensure a balance of the two interventions within each stratum.

### Concealment mechanism {16b}

The allocation sequence is implemented via sequentially numbered, opaque, sealed envelopes that are concealed until the interventions are assigned to a participant.

### Implementation {16c}

The study statistician generates the allocation sequence. Study interventionists enroll participants and, based on opening the envelope, assign participants to interventions.

## Assignment of interventions: blinding

### Who will be blinded {17a}

Research assistants are masked to randomization, and participants do not know the assigned group until after baseline measures are completed.

### Procedure for unblinding if needed {17b}

Unmasking is not needed.

## Data collection and management

### Plans for assessment and collection of outcomes {18a}

All staff involved in data collection must demonstrate competence in administering all measures. The research assistants collecting the data are masked to the participants’ intervention assignment. The research assistants review all assessment data for accuracy and completion. Participants are immediately re-contacted to provide missing data or to clarify responses. Loss to follow-up and missing and incomplete data are monitored closely to solve potential issues of missing data before there is a substantial impact on the results.

### Plans to promote participant retention and complete follow-up {18b}

To minimize loss to follow-up, at each data collection visit, participants are scheduled by phone, sent written reminders, and called the day before. Missed visits are rescheduled and followed up. Costs for transportation and childcare are provided, or alternatively, home visits are arranged for participants with repeatedly missed assessments. Honoraria are provided to promote retention: $25 for visits at study entry, 16 weeks, and 6 weeks postpartum visit; $15 per quarterly visit until conception; and $50 for the primary outcome assessment at 26 weeks’ gestation. As a retention tool, women in both groups also receive quarterly newsletters with basic information about preconception health and wellness.

### Data management {19}

Research Electronic Data Capture (REDCap) is used for storing study outcome measurement data. A customized internal study tracking system is used to track enrollment and scheduling of visits. Both systems require a login identification and password in order to gain access to the data. Range checks are built into the data collection procedures to alert staff to data that should be clarified. Error checking and preliminary analyses of all data are done to ensure accuracy. Electronic data files are backed up; a copy is stored offsite at both locations to protect against loss or damage. The destruction of any paper files will be at least 7 years from the termination of the study and will be authorized by the PI.

### Confidentiality {27}

Participant identification numbers are used to track questionnaires and data collection documents. A password-protected file is maintained that associates the participant’s name with the participant’s study identification number. Access to electronic data is password-protected and restricted to the study team. Paper data are stored in a locked file cabinet. Paper data may be removed for the purpose of coding, data entry, or auditing only. When taking participant files to intervention visits and assessments, files are transported in a locked box. Upon reaching the destination, these boxes are brought into the building or residence with the interventionists. Also, interventionists’ files identify participants by first name and last initial only. Participant home addresses are not included in the files. The study’s research coordinators in California and Rhode Island work closely with the statistician and data manager to ensure the secure exchange and storage of all project databases and questionnaires. Data exchanged between study sites are de-identified, encrypted, and password-protected.

### Plans for collection, laboratory evaluation, and storage of biological specimens for genetic or molecular analysis in this trial/future use {33}

As noted above, participants are asked to give explicit consent for the DNA and RNA collection and use, and future research of data and samples. For DNA and RNA, the collected samples sit in the collection tube at room temperature for 2 h then are placed in a − 20 °C freezer for the first 24 h before moving to − 80 °C freezer.

## Statistical methods

### Statistical methods for primary and secondary outcomes {20a}

A multiple logistic regression analysis will be used to examine the effect of the treatment group on the proportion of women who develop GDM. The model will include site and covariates to adjust for pre-randomization variables that may relate to the outcome, including parity, age, education, income, smoking, race/ethnicity, BMI, and time since last pregnancy. The effects of the pre-pregnancy weight loss intervention on weight, eating, activity, and physiologic outcomes will be examined using a linear mixed model with fixed effects for treatment condition (the between-groups factor), time (baseline, 16 weeks, 26 weeks’ gestation), site, and baseline covariates. Linear regression and logistic regression analyses will be performed to address the possible effects of the intervention on cases of excessive GWG, gestational hypertension, cesarean delivery, and large for gestational age at birth and 6 weeks, with site and baseline covariates entered in the models. A multiple linear regression analysis will also be used to examine the effect of the treatment group on composite scores of adverse maternal/neonatal outcomes, and logistic regressions will be used to examine separate effects on offspring obesity and odds of exceeding the National Academy of Science guidelines, including the same covariates described above.

### Interim analyses {21b}

The trial has no interim analyses or stopping rules.

### Methods for additional analyses (e.g., subgroup analyses) {20b}

Generalized logistic models will be used to examine the relationships among pre-pregnancy changes in weight, eating, activity, and physiology, and GDM recurrence; we will follow the approaches outlined by Kraemer et al. [164] to explore the potential mediators of the treatment outcome.

### Methods in analysis to handle protocol non-adherence and any statistical methods to handle missing data {20c}

Under intention-to-treat principles, all participants with confirmed pregnancy will be included in the primary analysis. If study measured OGTT results are not available, provider assessments will be used (based on chart abstraction). Missing data related to outcomes will be evaluated to assess whether the missing mechanism may be ignorable or non-ignorable [165–167]. If the missing data mechanism is judged to be ignorable, where appropriate, analyses involving mixed models may be used such that all existing values are analyzed, and no observations are deleted due to missing values. Alternatively, multiple imputations may be carried out to create several complete data sets. For each complete data set, overall tests of interest for the outcome will be conducted and the results of each combined to create a single test result. For completeness, a pattern missing analysis will be conducted to investigate non-ignorable missingness. If the missing data mechanism is likely to be non-ignorable, multiple imputations can be conducted using a version of the approximate Bayesian bootstrap based on distance-based selection criteria [168]. Sensitivity analyses under various assumptions regarding the missing data will be conducted to confirm the robustness of the results.

### Plans to give access to the full protocol, participant level-data {31c}

Upon publication of the study’s pre-specified outcomes, a de-identified version of the database will be made available upon reasonable request to the PI.

## Oversight and monitoring

### Composition of the coordinating center and trial steering committee {5d}

The primary decision-making body of this study is the investigative team comprising the principal investigator (PI, Phelan), the Miriam Hospital site PI (Wing), and the co-investigators. The PI and site PI are responsible for the overall management of the study. They coordinate the operations of the study, review issues that arise in the conduct of the study in between investigative team deliberations, and bring issues to the investigative team for decision. The PI (Phelan) serves as the liaison with the funding body, including submission of annual reports and providing overall management of the fiscal and administrative operations, and is also responsible for the study coordination and implementation at the Cal Poly site. The site PI (Wing) is responsible for the study coordination and implementation at the Miriam Hospital site.

The project coordinators (PCs) at each site are responsible for the day-to-day operations of the study, including recruitment, data collection processes, and intervention process. The PCs also coordinate IRB revisions and data monitoring reports and document completion of the trainings. The PCs work closely with each site’s budget analyst, research office, and investigators to ensure staff workload and progress are aligned with the budget. Research assistants (RAs) are responsible for recruiting and screening the participants, obtaining informed consent with participants, and scheduling and conducting follow-up assessments. Interventionists at each site are responsible for the treatment implementation. The data manager creates the data tracking system and supervises the development of the study’s REDCap surveys.

### Composition of the data monitoring committee, its role, and reporting structure {21a}

The trial includes two external safety officers with expertise in clinical trial weight control research and maternal/fetal health. Twice per year, safety officers review the reports of recruitment, retention, fidelity, and safety information on all participants, including the number of pregnancies before the 4-month intervention is over, number of injuries due to physical activity, number of miscarriages, and other serious adverse events. Weekly internal investigator meetings also occur to review the recruitment, attendance, retention, and safety data on an ongoing basis.

### Adverse event reporting and harms {22}

Adverse events (AEs) include any event that causes or increases the risk of harm to the participant or others. Serious adverse events (SAE) include any event that results in death, inpatient hospitalization or prolongation of existing hospitalization, a persistent or significant disability or incapacity, or a congenital anomaly or birth defect. A fatality, including fetal, is reported within 24 h. AEs reported at core assessment visits or informally at any time are evaluated by the research team and the investigators to determine if they are unanticipated problems involving risk to subjects and others or not. The participant’s situation is also assessed with regard to study and/or intervention continuation. Any SAEs are recorded by the research coordinator, reported to the PI and investigative team, the safety officers, and the IRB.

### Frequency and plans for auditing trial conduct {23}

The trial includes close monitoring by the PI/Co-Is, IRBs, and two external safety officers. Annual progress reports are provided to the sponsor and IRBs. Sponsor or other external site visitor audits are not planned.

### Plans for communicating important protocol amendments to relevant parties (e.g., trial participants, ethical committees) {25}

Any changes to the eligibility criteria, outcomes, or analyses are reviewed by the IRB and updated in ClinicalTrials.gov.

### Dissemination plans {31a}

The results of the trial will be presented at professional conferences and local community events and shared via ClinicalTrials.gov and formal publications and furthermore to the general public through social media outlets. A summary of the primary outcome findings will be created in English and Spanish and shared with the study participants.

## Discussion

GDM is recognized as a major adverse perinatal outcome and has been linked with a range of maternal and child complications and poor outcomes, including long-term development of type 2 diabetes. Recurrent GDM affects about 66–80% of women with obesity [19, 57, 58] and increases the number of maternal and child health risks significantly [18–27]. Additionally, identification and treatment of GDM exact a high cost to the health care system [169–171]. Preventing GDM and its recurrence has been identified as a national health priority [172, 173].

To date, efforts to prevent GDM have focused primarily on interventions occurring during pregnancy, and these have met with limited success [54, 60–65]. Promising, preliminary research from epidemiologic and retrospective bariatric surgery studies suggests that reductions in body weight before pregnancy may hold the key to the prevention of GDM and its recurrence [33–39]. Emergent research suggests that it is feasible to recruit women before pregnancy and promote significant weight loss prior to conception [40, 41]. A lifestyle intervention before pregnancy in women with prior GDM may capitalize on a “teachable moment” when women appear more motivated to engage in behavior changes to prevent the recurrence of GDM in a subsequent pregnancy [42–45]. However, a fully powered trial to test the effects of maternal lifestyle intervention before pregnancy to reduce body weight and prevent GDM recurrence has never been conducted.

The Gestational Diabetes Prevention/Prevención de la Diabetes Gestacional is the first trial designed to determine whether preconception weight loss can prevent GDM recurrence in a diverse population of Hispanic and non-Hispanic women who are disproportionately impacted by GDM [57, 174, 175]. The SCT-based intervention is uniquely designed to capitalize on the potential “teachable moment” for women with prior GDM who report high motivation to change behaviors to prevent GDM recurrence and protect the health of their future baby [42–45]. The study’s battery of measures before pregnancy will provide the first comprehensive picture of how maternal weight, diet, activity, and physiology *before pregnancy* impact GDM, insulin resistance, and cardiometabolic health. The study is providing a new scientific framework for future pre-pregnancy trials by informing optimal methods for reaching women before pregnancy and the best timing, content, and duration of effective GDM prevention interventions. The American College of Obstetricians and Gynecologists (ACOG), the National Academy of Medicine, and other governmental bodies and researchers [172, 173, 176–182] have identified the interconception interval as one of the best potential times for weight control intervention to minimize the risk of a GDM and its recurrence. If successful, the results of this study will yield a novel, empirically based intervention that can be used during the interconception period to prevent GDM.

## Trial status

Protocol version 1.0; February 15, 2021. Recruitment was initiated in August 2016, and the approximate date for completion considering delays due to the COVID-19 pandemic is December 2021.

## Abbreviations

NAS: National Academy of Science
BMI: Body mass index
OGTT: Oral glucose tolerance test
